# Supplementary material for: Bacterial and Genetic Features of Raw Retail Pork Meat: Integrative Analysis of Antibiotic Susceptibility, Whole-Genome Sequencing, and Metagenomics
Source: Antibiotics (Basel). 2024 Jul 26;13(8):700. doi: 10.3390/antibiotics13080700 (PMC11350697; doi:10.3390/antibiotics13080700)
Supplement: Supplementary file 1 [file antibiotics-13-00700-s001.zip › antibiotics-3104401-supplementary.pdf]

Supplementary Material

Table S1. Captured demographic data collected from the sampled stores five supermarkets and five butcheries)

Collection date	Sample ID	Store ID	Number of cash registers	Sell-by date	Packaging date	Frozen meat label	Final processing in store	Organic meat	Free range meat	Antibiotic free meat	Price per kilogram^	Other	*ESCCO* count	*SAL* spp. count	*ENT spp.* count	*CAM* spp. count
4-Jan-22	PC1	S1	>= 26	9-Jan-22	31-Dec-21	No	Yes	No	No	Unknown	ZAR 109,99 / USD 6,72	Pork 360 quality sticker	Absent	Absent	Absent	Absent
4-Jan-22	PC2	S2	6-10	4-Jan-22	Unknown	No	Unknown	No	No	Unknown	ZAR 119,99 / USD 7,33	Kinder to sows sticker	Absent	Absent	Absent	Absent
4-Jan-22	PC3	S3	6-10	7-Jan-22	Unknown	No	Unknown	No	No	Unknown	ZAR 99,99 / USD 6,05	Sow friendly sticker	Absent	Absent	16	Absent
4-Jan-22	PC4	S4	6-10	5-Jan-22	2-Jan-22	No	Yes	No	No	Unknown	ZAR 89,99 / USD 5,50	N/A	Absent	Absent	1	Absent
4-Jan-22	PC5	S5	11-15	8-Jan-22	Unknown	No	Yes	No	No	Unknown	ZAR 85,99 / USD 5,25	N/A	Absent	Absent	Absent	Absent
4-Jan-22	PC6	B1	1-5	6-Jan-22	2-Jan-22	No	Yes	No	No	Unknown	ZAR 79,99 / USD 4,89	N/A	Absent	Absent	Absent	Absent
4-Jan-22	PC7	B2	6-10	7-Jan-22	2-Jan-22	No	Yes	No	No	Unknown	ZAR 89,99 / USD 5,50	N/A	Absent	Absent	Absent	Absent
4-Jan-22	PC8	B3	1-5	Unknown	3-Jan-22	No	Yes	No	No	Unknown	ZAR 90,00 / USD 5,50	N/A	Absent	Absent	Absent	Absent
4-Jan-22	PC9	B4	1-5	Unknown	4-Jan-22	No	Yes	No	No	Unknown	ZAR 89,90 / USD 5,50	N/A	20	Absent	Absent	Absent
4-Jan-22	PC10	B5	1-5	10-Jan-22	3-Jan-22	No	Yes	No	No	Unknown	ZAR 96,90 / USD 5,92	N/A	Absent	Absent	3	Absent

PC = Pork chop; S = Supermarket; B = Butchery; ESCCO = *Escherichia coli*; SAL = *Salmonella;* ENT = *Enterococcus*; CAM = *Campylobacter*; ZAR = South African Rand; USD = United States dollar.

^The average exchange rate for 2022 was used to convert the cost of meat per kilogram from ZAR to USD (i.e. 16.37 ZAR = 1 USD).

Table S2. Summary of antimicrobial resistance categories by number of open reading frame genes from 10 raw retail meat samples.

AMR categories	Supermarkets (n = Annotated ORFs)	Butcheries (n = Annotated ORFs)	Overall (n = Annotated ORFs)
Aminoglycoside	4	2	6
Bacitracin	1	1	2
Beta-lactam	**14**	1	15
Fluoroquinolone	1	1	2
Fosfomycin	0	1	1
Glycopeptide	5	1	6
MLS	8	2	10
Multidrug	**18**	**3**	21
Mupirocin	0	1	1
Peptide	2	1	3
Phenicol	3	0	3
Sulphonamide	1	0	1
Tetracycline	**10**	**8**	18

AMR = Antimicrobial resistance; ORF = Open reading frame; MLS = Macrolides, lincosamides and streptogramins.

Table S3. Summary of antimicrobial resistance mechanisms by number of ORF genes from 10 raw retail meat samples.

AMR resistance mechanisms	Supermarkets (n = Annotated ORFs)	Butcheries (n = Annotated ORFs)	Overall (n = Annotated ORFs)
Antibiotic efflux	30	13	43
Antibiotic inactivation	19	3	22
Antibiotic target alteration	14	6	20
Antibiotic target protection	1	0	1
Antibiotic target replacement	3	0	3
Reduced permeability to antibiotic	2	0	2
Resistance absence	1	0	1

AMR = Antimicrobial resistance; ORF = Open reading frame

Table S4. Summary of virulence factor category by number of ORF genes from 10 raw retail meat samples.

VF category	Supermarkets (n = annotated ORFs)	Butcheries (n = annotated ORFs)	Overall (n = annotated ORFs)
Adherence	39	13	42
Antimicrobial activity/Competitive advantage	3	2	5
Biofilm	6	1	7
Effector delivery system	16	3	19
Exoenzyme	1	0	1
Exotoxin	2	0	2
Immune modulation	10	2	10
Invasion	7	0	7
Motility	11	2	13
Nutritional/Metabolic factor	11	4	15
Post-translational modification	2	0	2
Regulation	4	1	5
Stress survival	4	0	4

VF = Virulence factor

Table S5. Summary of toxin gene description by number of ORF genes from 10 raw retail meat samples.

**Toxin gene description**	**Supermarkets (n = Annotated ORFs)**	**Butcheries (n = Annotated ORFs)**	**Overall (n = Annotated ORFs)**
AcrB/AcrD/AcrF family	5	4	9
Capsule assembly protein Wzi	1	0	1
Dual specificity phosphatase, catalytic domain	1	0	1
Endonuclease/Exonuclease/phophatase family	1	0	1
Insulinase (Peptidase family M16)	9	0	9
Outer membrane protein	9	1	10
PapC N-terminal domain	1	0	1
Peptidase M16 inactivate domain	1	0	1
Presequence protease [EC:3.4.24.-]	1	0	1
Probable enterotoxin B	1	0	1
Probable enterotoxin D	1	0	1
Putative toxin 46	1	0	1
S-type Pyocin	1	0	1
S1/P1 Nuclease	1	0	1
Putative hemolysin	0	1	1

Table S6. Demographic data collected from the sampled stores (five supermarkets and five butcheries)

NATIONAL INSTITUTE FOR COMMUNICABLE DISEASES
Division of the National Health Laboratory Service

Date of sample collection: ______________________________

Sample collection done by: ______________________________

Sample unique identifier: ______________________________

Store unique identifier: ______________________________

1. Total number of cash registers in-store (a surrogate measure of store volume)

1 to 5 ☐ 6 to 10 ☐ 11 to 15 ☐ 16 to 20 ☐ 21 to 25 ☐ ≥26 ☐

2. "Sell-by" and packaging date of meat

"Sell-by" date: ______________________________

Packaging date: ______________________________

3. "May contain previously frozen meat" label

Yes ☐ No ☐

4. Final processing in store

Yes ☐ No ☐ Unknown ☐

5. Organic meat

Yes ☐ No ☐ Unknown☐

6. Free range meat

Yes ☐ No ☐ Unknown ☐

7. Antimicrobial free meat

Yes ☐ No ☐ Unknown ☐

8. Price per kilogram: R ______________________________

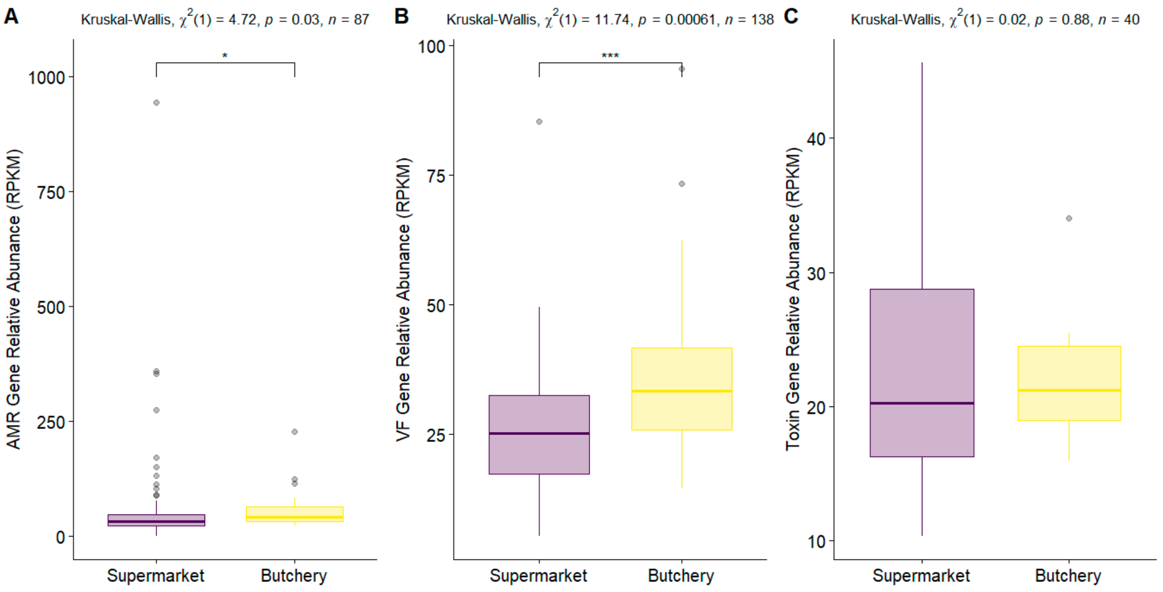

Figure S1. Relative abundance of ARGs, VFs, and toxin genes of 10 raw meat samples (normalized as reads per kilobase of reference sequence per million mapped sample reads (RPKM), grouped by source).

Pork meat source medians are shown as vertical lines and the interquartile ranges (25th and 75th percentiles) as boxplot hinges. The horizontal lines represent the highest and lowest values. Outliers are represented as grey dots. The significance levels are '*' = ≤ 0.05; '**' ≤ 0.01; '***' = ≤ 0.001.

A Kruskal-Wallis test from the 'rstatix' package (v0.7.2) was applied to study whether the differences between Supermarket and Butchery meat samples were significant. There were statistically significant differences in the AMR and VF gene relative abundance between the different meat samples (p=0.03 and p =0.00061, respectively). However, there is no statistically significant difference in the toxin gene relative abundance between the different meat source.

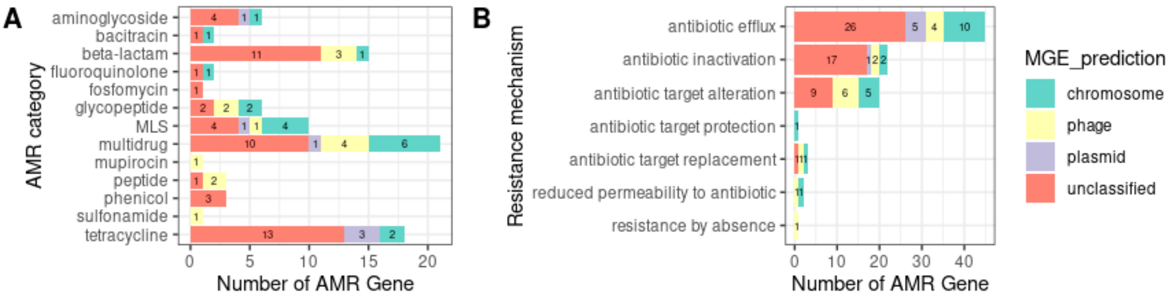

Figure S2. Mobile genetic element prediction of ARGs of all raw meat samples from both supermarket and butchery groups. Unclassified MGE predictions are genes with unknown mobile genetic element information.

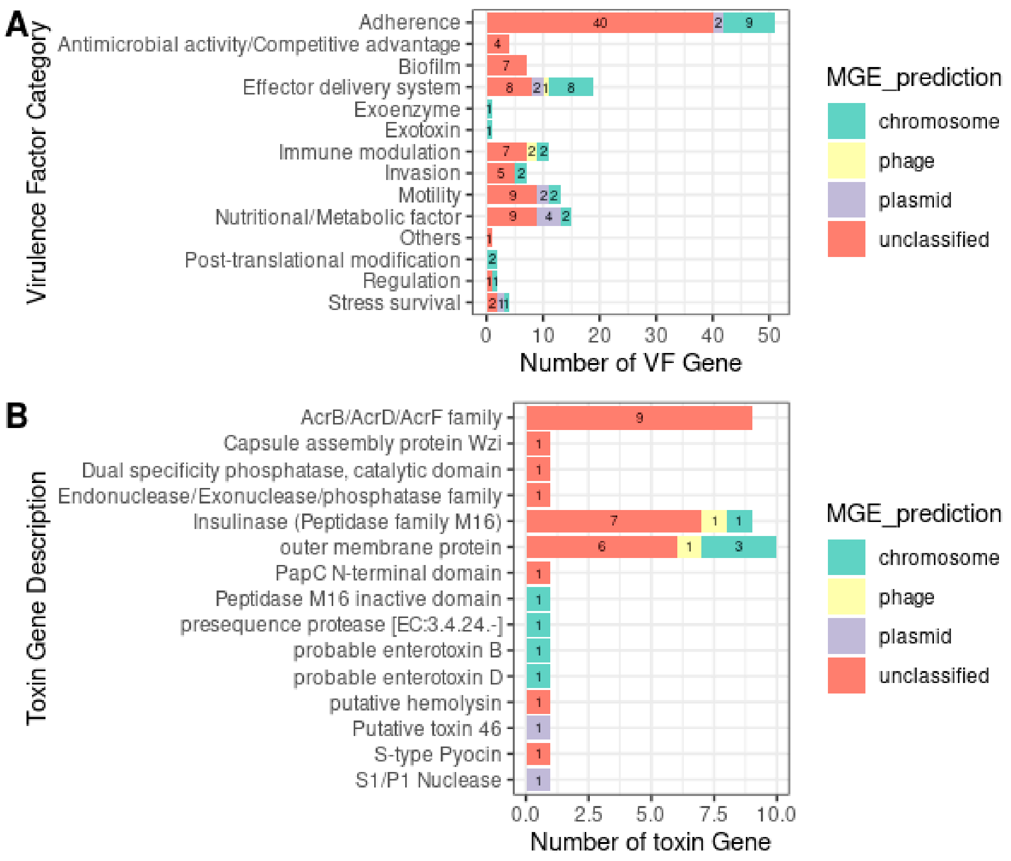

Figure S3. Mobile genetic element prediction of VF and toxin genes of all raw meat samples from both supermarket and butchery groups.
